# Descending projections from auditory cortex to excitatory and inhibitory cells in the nucleus of the brachium of the inferior colliculus

**DOI:** 10.3389/fnsys.2014.00188

**Published:** 2014-10-06

**Authors:** Jeffrey G. Mellott, Martha E. Bickford, Brett R. Schofield

**Affiliations:** ^1^Department of Anatomy and Neurobiology, Northeast Ohio Medical UniversityRootstown, OH USA; ^2^Department of Anatomical Sciences and Neurobiology, University of LouisvilleLouisville, KY USA

**Keywords:** medial geniculate nucleus, ascending, GABA, corticofugal, modulation

## Abstract

Descending projections from the auditory cortex (AC) terminate in subcortical auditory centers from the medial geniculate nucleus (MG) to the cochlear nucleus, allowing the AC to modulate the processing of acoustic information at many levels of the auditory system. The nucleus of the brachium of the inferior colliculus (NBIC) is a large midbrain auditory nucleus that is a target of these descending cortical projections. The NBIC is a source of several auditory projections, including an ascending projection to the MG. This ascending projection appears to originate from both excitatory and inhibitory NBIC cells, but whether the cortical projections contact either of these cell groups is unknown. In this study, we first combined retrograde tracing and immunochemistry for glutamic acid decarboxylase (GAD, a marker of GABAergic cells) to identify GABAergic and non-GABAergic NBIC projections to the MG. Our first result is that GAD-immunopositive cells constitute ~17% of the NBIC to MG projection. We then used anterograde labeling and electron microscopy to examine the AC projection to the NBIC. Our second result is that cortical boutons in the NBIC form synapses with round vesicles and asymmetric synapses, consistent with excitatory effects. Finally, we combined fluorescent anterograde labeling of corticofugal axons with immunochemistry and retrograde labeling of NBIC cells that project to the MG. These final results suggest first that AC axons contact both GAD-negative and GAD-positive NBIC cells and, second, that some of cortically-contacted cells project to the MG. Overall, the results imply that corticofugal projections can modulate both excitatory and inhibitory ascending projections from the NBIC to the auditory thalamus.

## Introduction

The discovery of extensive projections from the auditory cortex (AC) to brainstem auditory nuclei below the inferior colliculus (IC) (Feliciano et al., 1995) substantially broadened our view of the corticofugal system (reviewed by Schofield, 2010, 2011; Malmierca and Ryugo, 2011). Corticofugal projections terminate in auditory centers from the medial geniculate nucleus (MG) to the cochlear nucleus, presumably allowing the AC to modulate processing of acoustic information at many levels of the auditory system. Physiological studies have implicated these cortical projections in a wide range of functions, including the tuning of cells for a variety of stimulus parameters (e.g., frequency, amplitude, duration; Popelár et al., 2003; Suga, 2008; Xiong et al., 2009; He and Yu, 2010), stimulus-specific adaptation and sensitivity to pitch and spatial cues (Jen et al., 1998; Nakamoto et al., 2008, 2010; Anderson and Malmierca, 2013) and adaptation to changing spatial cues (Bajo et al., 2010). Identification of the subcortical circuits contacted by cortical axons is an important step for understanding the mechanisms of corticofugal effects and characterizing their broader functions.

The present study focuses on a subcortical area, the nucleus of the brachium of the inferior colliculus (NBIC), that has been largely ignored in recent examinations of the corticofugal system. The NBIC is a large midbrain auditory nucleus associated with a prominent fiber tract, the brachium of the IC. This brachium contains the axons of IC cells that project to the MG as well as many descending axons that originate in AC and travel to the IC (Diamond et al., 1969; Saldaña et al., 1996). The fiber tract extends from the lateral part of the IC rostrally through the lateral midbrain tegmentum to reach the caudal end of the MG. A large number of cells are distributed along this pathway; many cells are concentrated in the medial part of the fiber tract, although the latter, fiber-rich area contains a substantial number of neurons (e.g., Kudo et al., 1984; Morest and Oliver, 1984; King et al., 1998).

The NBIC has been implicated in a variety of functions, including orienting responses, auditory attention and multimodal processing. These functions are served by inputs to the NBIC from numerous auditory centers, most notably the IC and the AC, and substantial projections from the NBIC to the MG and the superior colliculus (Calford and Aitkin, 1983; Kudo et al., 1984; LeDoux et al., 1985; Thiele et al., 1996; Jiang et al., 1997; King et al., 1998). NBIC cells respond to acoustic stimuli and can be sensitive to azimuth and elevation cues that are used for sound localization (Aitkin and Jones, 1992; Schnupp and King, 1997; Slee and Young, 2013). The NBIC receives input from somatosensory and visual nuclei, and appears to play a role in integration of multiple sensory modalities (Berkley et al., 1980; Flink et al., 1983; Itoh et al., 1984; Wiberg and Blomqvist, 1984; Redgrave et al., 1987; Doubell et al., 2000). The multimodal nature of NBIC is reflected in its projections to the medial geniculate nucleus, where NBIC axons terminate primarily in the medial, dorsal and suprageniculate subdivisions (i.e., outside the ventral MG, the main relay for lemniscal information; Calford and Aitkin, 1983; Kudo et al., 1984). These subdivisions are part of the extralemniscal pathway and include cells with multimodal responses. They may be particularly important for detecting change and analyzing stimulus context (discussed in Anderson and Linden, 2011).

Glutamate and GABA are the major effectors of ascending auditory inputs to the MG. Electrical stimulation of the brachium of the IC produces excitation and inhibition of MG cells that can be eliminated by pharmacological blockade of glutamate and GABA receptors in the MG (Peruzzi et al., 1997; Bartlett and Smith, 1999, 2002; Smith et al., 2007). Anatomical studies have identified GABAergic and non-GABAergic projections to the MG from the IC in several species (cats: Winer et al., 1996; rats: Peruzzi et al., 1997; guinea pigs: Mellott et al., 2014). A GABAergic projection from the NBIC to the MG has also been identified, but thus far has been described only in rats (Peruzzi et al., 1997). In the first part of the present study, we document a GABAergic projection from the NBIC to the MG in guinea pigs.

The NBIC is also a target of descending projections from the AC (Diamond et al., 1969; Andersen et al., 1980). These projections originate from a wide span of cortical areas, including primary AC (Saldaña et al., 1996; Budinger et al., 2006) as well as other AC areas (Winer et al., 1998; Kimura et al., 2004). To our knowledge, there are no studies comparing relative strengths of projections to NBIC from different cortical areas, but the studies cited above (based on anterograde tracing or degeneration) clearly indicate projections from areas believed to have distinct functions (e.g., primary vs. non-primary areas). Whether the cortical projections contact NBIC cells that project to the MG is unknown. In the second part of the present study, we describe evidence that auditory cortical axons terminate on cells in the NBIC and that the targets include GABAergic and non-GABAergic cells that project to the MG. These results imply that auditory corticofugal projections could activate, or modulate, both excitatory and inhibitory ascending projections from the NBIC to the auditory thalamus.

## Materials and methods

All procedures were conducted in accordance with the Institutional Animal Care and Use Committee and NIH guidelines. Results are described from 15 adult pigmented guinea pigs (Elm Hill Labs; Chelmsford, MA, USA) of either gender weighing 317–1058 g. Efforts were made to minimize the number of animals and their suffering.

### Surgery

Each animal was anesthetized with halothane or isoflurane (4–5% for induction, 1.75–3% for maintenance) in oxygen. The head was shaved and disinfected with Betadine (Purdue Products L.P., Stamford, CT, USA). Atropine sulfate (0.08 mg/kg i.m.) was given to minimize respiratory secretions and Ketofen (ketoprofen, 3 mg/kg i.m.; Henry Schein, Melville, NY 11747, USA) was given for post-operative pain management. Ophthalmic ointment (Moisture Eyes PM, Bausch & Lomb, Rochester, NY, USA) was applied to each eye to protect the cornea. The animal's head was positioned in a stereotaxic frame. Body temperature was maintained with a feedback-controlled heating pad. Sterile instruments and aseptic techniques were used for all surgical procedures. An incision was made in the scalp and the surrounding skin was injected with Marcaine (0.25% bupivacaine with epinephrine 1:200,000; Hospira, Inc., Lake Forest, IL, USA), a long-lasting local anesthetic. A craniotomy was made over the desired target coordinates using a dental drill. In animals that received tracer deposits in multiple areas (left and right MG or an MG and the AC), all deposits were done during a single surgery. Following tracer injections, Gelfoam (Harvard Apparatus, Holliston, MA, USA) was placed in the craniotomy and the scalp was sutured. The animal was then removed from the stereotaxic frame and placed in a clean cage. The animal was monitored until it could walk, eat and drink without difficulty.

### Tracers

Five fluorescent tracers were used for retrograde labeling of NBIC cells from the MG: red fluorescent RetroBeads (“red beads,” Luma-Fluor, Inc., Naples, FL, USA; injected without dilution); green fluorescent RetroBeads (“green beads,” LumaFluor; injected without dilution); Fast Blue (FB, 5% in water; EMS-Chemie GmbH, Gross Umstadt, Germany), FluoroGold (4% in sterile water; FluoroChrome, Inc., Englewood, CO, USA) and fluorescein dextran (FD, 10% in saline, 10 k molecular weight; Invitrogen, Carlsbad, CA, USA) mixed in equal proportion with 4% FG (FG/FD). The use of this variety of tracers provides numerous advantages not available with a single tracer. All the tracers used in the present study are known to be sensitive retrograde markers (Schofield et al., 2007; Schofield, 2008). In addition, FB and FG diffuse readily, facilitating large injections and attempts to maximize the spread of injections to include as much of the target as possible. In contrast, red or green beads show very limited diffusion. This facilitates very small injections that are still informative because of the high sensitivity of the beads. The dextrans are less sensitive as retrograde tracers (in many pathways they label fewer cells than other tracers with a similar injection size), but often provide the most extensive labeling of dendrites. The extent of dendritic labeling varies with the pathway (e.g., Schofield and Cant, 1996; Bajo and Moore, 2005; Schofield et al., 2006), but still provides some of the best opportunities for identifying contacts between labeled cells and labeled axons (e.g., Coomes Peterson and Schofield, 2007). Stereotaxic coordinates were used to target specific MG subdivisions. Large injections were made with a microsyringe (1 μl; Hamilton, Reno, NV, USA) (Table 1). Each syringe was dedicated to a single tracer or specific mixture. Small injections were made with a micropipette (tip inside diameter 25 μm) attached to a Nanoliter Injector (World Precision Instruments, Sarasota, FL, USA).

**Table 1:** **Injections into the MG or AC**.

**Case**	**MG injections**							**AC injections**			
	**Side**	**Tracer**	**Total volume**	**MGv**	**MGd**	**MGsg**	**MGm**	**Side**	**Tracer**	**No. of deposit sites**	**Total volume (μl)**
GP636	R	FG	0.05 μl	x	x	x	x				
GP640	L	RB	0.4 μl	x	x	x	x				
GP640	R	FB	0.08 μl	x	x	x	x				
GP689	L	RB	69 nl			(x)	x				
GP689	R	GB	69 nl				x				
GP692	L	FG	0.05 μl	x	x	x	x				
GP693	L	RB	46 nl		x						
GP695	L	RB	27.6 nl	x							
GP696	L	RB	27.6 nl			x					
GP698	L	RB	18.4 nl			x					
GP718	L	FG	0.05 μl		x			L	FR	9	2.4
GP719	L	FG	0.05 μl	x				L	FR	12	3.2
GP721	L	FG	0.08 μl	x	x	x	x	L	FR	15	4.0
GP722	L	FG/FD	0.08 μl			x	x	L	FR	15	3.6
GP726	L	FG	0.08 μl	x	x			L	FR	17	4.6
GP363^*^								L	FD	32	6.4
GP391^*^								L	FR	5	0.9

*Summary of injection parameters and distribution of deposit sites for tracer injections. Fluorescent tracers were deposited into left (L) or right (R) medial geniculate nuclei (MG) and/or into the left auditory cortex (AC). Total volume = volume of tracer deposited within the target on the indicated side. Volumes indicated in nanoliters (nl) represent injections made with a Nanoliter Injector (see text); volumes indicated in microliters (μl) were made with a 1 μl microsyringe or, for some injections into the AC, with a 10 μl microsyringe. For each MG injection, the subdivisions that were included in the deposit sites are indicated: “x” indicates substantial deposit within the subdivision, “(x)” indicates only minor encroachment of the tracer into that subdivision. An empty space indicates no significant spread of tracer into that subdivision. In Case GP692, the tracer spread medial and ventral to the MGm; none of the other cases had significant tracer spread outside the MG. FB, Fast Blue; FG, FluoroGold; FG/FD, 1:1 mixture of FG and fluorescein dextran; GB, green beads; RB, red beads*.

* , *animals that were analyzed with electron microscopy*.

Fluorescent dextrans have proven to be superb anterograde tracers for labeling AC axons extending to subcortical sites in guinea pigs (cochlear nucleus: Schofield and Coomes, 2005a,b; IC: Nakamoto et al., 2013a,b; superior olivary complex: Coomes and Schofield, 2004; pontomesencephalic tegmentum: Schofield and Motts, 2009). FD and FluoroRuby (FR; tetramethylrhodamine dextran, 10 k molecular weight, 10% in sterile saline) were used for anterograde tracing from the AC. Each tracer was injected with a 1 μl Hamilton microsyringe dedicated to use with that tracer. Tracer was deposited into the left AC of 5 of the animals with MG injections (Table 1). Two additional animals received injections of tracer into the AC for subsequent analysis with electron microscopy (Table 1). In all cases, tracer was deposited in a grid-like array (9–32 deposit sites) centered on core auditory fields using the pseudosylvian sulcus as a surface landmark (primary AC and the dorsocaudal field; see Wallace et al., 2000, 2002). Volumes of 0.1–0.2 μl were deposited at each site for a total volume of 2.4–6.4 μl in a given animal.

### Perfusion and tissue processing

#### Light microscopy

Five to twenty days after surgery, the animal was deeply anesthetized with isoflurane and perfused transcardially with Tyrode's solution (a bicarbonate-buffered Ringer's solution; http://en.wikipedia.org/wiki/Tyrode's_solution), followed by 250 ml of 4% paraformaldehyde in 0.1 M phosphate buffer, pH 7.4 and then 250 ml of the same fixative with 10% sucrose. The brain was removed from the skull and stored at 4°C in fixative with 25–30% sucrose. The following day the cerebellum was removed and the cerebral cortex was separated from the brainstem and thalamus (separating the tissue simplifies processing and minimizes the amount of immunochemical reagents needed for staining and, in relevant cases, for electron microscopy). The brainstem was further trimmed with transverse cuts at the rostral end of the thalamus and posterior to the superior olive. Each piece of tissue was frozen and cut on a sliding microtome into sections 40 or 50 μm thick that were collected serially in six sets. The cortex was cut in the transverse plane; the brainstem/thalamus was cut in transverse, sagittal or horizontal planes.

One series of brainstem sections was split into rostral and caudal sets so that sections through the thalamus could be stained for cytochrome oxidase activity to identify MG subdivisions (Anderson et al., 2007) and sections through the midbrain could be stained with antibodies to brain nitric oxide synthase (Table 2) to identify IC subdivisions (Coote and Rees, 2008) and facilitate identification of NBIC borders (personal observations). One or more additional series of sections were stained to identify putative GABAergic cells with antibodies to glutamic acid decarboxylase (GAD) (Nakamoto et al., 2013c). Briefly, the sections were pretreated with normal goat serum to limit non-specific labeling, then exposed (1–2 days at 4°C) to mouse anti-GAD polyclonal antibody (Table 2). The sections were treated with 1% biotinylated goat anti-mouse antibody and labeled with streptavidin conjugated to a fluorescent marker (AlexaFluor 488). In some cases with cortical injections, synaptic sites were stained in one series of sections by treatment with antibodies to synapsin 1 (Table 2), a synaptic marker. Specificity of the anti-synapsin 1 antibody was established by western blot (Abcam). The marker was visualized with goat anti-rabbit secondary antibody conjugated to AlexaFluor 750 (Table 2). For some series, NeuroTrace 435/455 Fluorescent Nissl Stain (Cat # N21479; Molecular Probes, www.lifetechnologies.com) was used as a Nissl counterstain. Stained sections were mounted on gelatin-coated slides, allowed to dry and coverslipped with DPX (www.sigmaaldrich.com).

**Table 2:** **List of main reagents used for immunochemistry**.

	**Host**	**Conjugated to**	**Working dilution**	Source^*^	**Catalog number**
**PRIMARY ANTIBODY**					
Anti-bNOS	Mouse		1:1000	Sigma	N2280
Anti-GABA	Rabbit		1:500–1:1000	Sigma	A2052
Anti-GAD67	Mouse		1:100–1:1000	Millipore	MAB5406
Anti-synapsin 1	Rabbit		1:500–1:1000	Abcam	Ab8
Anti-fluorescein	Goat	Biotin	1:1000	Vector	BA-0601
Anti-rhodamine	Goat	Biotin	1:1000	Vector	BA-0605
**SECONDARY ANTIBODY**					
Anti-Mouse	Goat	Biotin	1:100	Vector	BA-9200
Anti-Rabbit	Goat	AF750	1:100	Invitrogen	A21039
Anti-Rabbit	Goat	15 nm gold	1:25	Ted Pella	15727
**TAG**					
Streptavidin		AF488	1:100	Invitrogen	S-11223
Streptavidin		AF647	1:100	Invitrogen	S-21374
Streptavidin		Peroxidase	1:100	Jackson	016-030-084

*Abbreviations: bNOS, brain nitric oxide synthase; GAD, glutamic acid decarboxylase; AF488, AF647, AF750: AlexaFluor fluorescent marker excited maximally by wavelength indicated by the number (in nm)*.

* *Sources: Abcam, www.abcam.com; Jackson ImmunoResearch, www.jacksonimmuno.com; Invitrogen, www.lifetechnologies.com; Millipore, www.millipore.com; Sigma, www.sigmaaldrich.com; Ted Pella, www.tedpella.com; Vector, www.vectorlabs.com*.

In animals that received cortical injections, sections through AC were examined to ensure that the tracer did not penetrate the underlying white matter or deeper brain structures. All cases had extensive labeling of cells in the MG, confirming injection into the AC.

#### Processing for electron microscopy

Two animals that received tracer injections into the AC were processed for electron microscopy as described previously (Nakamoto et al., 2013b). Briefly, the animals were perfused after 11–18 days as described above except that the fixative was 2% glutaraldehyde plus 2% paraformaldehyde in 0.1 M phosphate buffer. The brain was stored in the fixative overnight and then sectioned on a Vibrotome (50 μm thick sections) in the sagittal plane. Tracer was revealed with biotinylated antibodies to fluorescein or rhodamine (Table 2) and streptavidin peroxidase, which was then used to precipitate diaminobenzidine (DAB). Some of the reacted sections were mounted on glass slides, counterstained for Nissl substance, and covered for light microscopic examination. A subset of sections with labeled axons were post-fixed in 2% osmium tetroxide, dehydrated in alcohols and embedded in Durcupan (Electron Microscopy Sciences, Fort Washington, PA, USA) between sheets of Aclar Embedding Film (Ted Pella, Inc. Redding, CA, USA). These sheets were examined in a light microscope and areas of the NBIC that contained labeled axons were trimmed out and mounted on a blank resin block. Ultrathin sections (silver-gold interference) were cut with a Leica UC6 ultramicrotome, collected on 300 mesh nickel grids and stained with antibodies to GABA to reveal GABAergic neurons (described in detail in Coomes et al., 2002). Briefly, grids holding thin sections were placed on rabbit anti-GABA antibodies (Table 2) overnight, then marked with secondary antibodies linked to 15 nm gold particles (Table 2). Sections were stained with 1% uranyl acetate and, in some cases, lead citrate.

### Data analysis

#### Light microscopy—brightfield and wide-field fluorescence

***Cytoarchitecture and injection sites***. The location and extent of each MG injection site was determined by comparison of the tracer deposit with borders of MG subdivisions identified in sections stained for cytochrome oxidase (Anderson et al., 2007). Injections into the cortex were examined to confirm that they were located in the AC (i.e., in temporal cortex between pseudosylvian and rhinal sulci; cf. Wallace et al., 2000, 2002) and did not extend into the subcortical white matter or other structures. The NBIC was identified by comparison with descriptions in cat (Kudo et al., 1984; Morest and Oliver, 1984) and ferret (King et al., 1998). Identification of the borders of the NBIC with the IC and with the MG were facilitated by both the bNOS and the GAD immunochemistry (see Results).

***Immunochemistry***. Immunostaining revealed GAD-immunoreactive (GAD+) cells and boutons in the NBIC. Immunopositive cells were labeled intensely and were readily distinguished from immunonegative cells. The GAD immunostain was also readily visible in tracer-labeled cells, making it straightforward to distinguish GAD+ vs. GAD-negative staining in the retrogradely labeled cells. Results from 8 injections (3 large and 5 small) that had robust immunostaining were used for quantitative analysis. Labeled cells in the NBIC were plotted with a Neurolucida reconstruction system (MBF Bioscience, Williston, VT, USA) attached to a Zeiss Axioplan II microscope (Carl Zeiss MicroImaging, Inc., Thornwood, NY, USA). For each case, every labeled cell was plotted in an evenly spaced series of sections (usually every sixth section) through the NBIC ipsilateral to the tracer deposit.

In some cases, the anti-GAD staining did not fully penetrate the tissue, resulting in a central layer in the section where GAD staining was absent. Data from these cases were plotted with the Neurolucida system and a 63× objective (NA = 1.4), with special attention to focusing on the center of the soma when plotting the symbol for a particular cell. This approach provides sufficient resolution in the z plane (section depth) to allow filtering of the data by depth. After the data were plotted, the X, Y, and Z coordinates of all markers from each subdivision of each tissue section were exported from Neurolucida to Microsoft Excel and sorted based on the Z coordinate. The depth of penetration of the GAD labeling was assessed under the 63× objective to determine the range of depths (measured from each surface of the section) where GAD staining was robust. This yielded 2 zones of data from each section (1 associated with each surface), and a central zone that was stained poorly or not at all with GAD. All markers in the central zone were excluded from further analyses. (Note: the central zone is typically 10–15 μm thick, given that the sections, which were cut at 40 or 50 μm thick, are usually 20–25 μm thick once dehydrated and coverslipped for microscopy).

In order to assess the relationship of cortical synapses to NBIC cells in our light microscopic experiments, we examine tracer-labeled cortical boutons in tissue stained with antibodies to the presynaptic synaptic marker synapsin 1. We counted double-labeled boutons across the NBIC in two sagittal sections, thus including samples from a wide rostro-caudal and medio-lateral expanse of the NBIC. The tissue was examined at high magnification (63× oil objective, NA = 1.4) to allow careful assessment of colocalization between the tracer label and the anti-synapsin label. In addition, analysis was limited to a region near the surface of the section where synapsin staining was robust. Our goal was to assess the proportion of likely cortical synapses that were associated with NBIC somas vs. those located in the neuropil, and thus likely to contact dendrites of the NBIC cells. By selecting only double-labeled boutons, we avoided false negative staining that could have arisen from incomplete penetration of the anti-synapsin immunochemicals. Given these constraints, all double-labeled boutons in the stained zone near the top surface of each tissue section were counted. This yielded 455 boutons. The boutons were classified as “perisomatic” if they appeared to be in close apposition to a NeuroTrace-labeled soma.

#### Structured illumination fluorescence microscopy

High resolution imaging of fluorescent structures was accomplished with structured illumination microscopy (Apotome 2 system) on a Zeiss AxioImager Z2 (www.zeiss.com/microscopy) controlled by Neurolucida software (version 11.03, MBF Bioscience). Most often, optical sections were collected at a spacing of 0.2 μm, over a total depth of 4–6 μm, although finer spacing and greater depths were employed as necessary. The system is equipped with a metal halide illuminator (X-Cite 120Q, Lumen Dynamics, www.ldgi.com) allowing for fluorescence analysis throughout the visible spectrum and in several infrared channels (excitation at 647 or 750 nm).

#### Electron microscopy

Ultrastructure was analyzed with two electron microscopes (EM). One EM is a JEOL JEM 100S transmission electron microscope with which areas of interest were photographed at 15,000–40,000 magnification with high-resolution film (Kodak SO-163; Kodak, Rochester, NY, USA). The negatives were scanned at 1200–2000 pixels/inch (ScanMaker 800, Microtek, Santa Fe Springs, CA, USA) to produce digital images. A second EM is a Phillips CM10 equipped with a digital camera for direct capture of digital images. Synapses were identified by the presence of vesicles in the presynaptic profile, a clear synaptic cleft and the presence of a postsynaptic density. Tracer-labeled profiles were readily identified by the presence of DAB precipitate. GABA-positive (GABA+) profiles were easily distinguished from GABA-negative profiles by a distinct difference in the density of overlying gold particles (Nakamoto et al., 2013a). GABA immunostaining was also readily distinguished from the DAB label (Nakamoto et al., 2013b).

### Figure production

Plots showing the distribution of labeled cells were created with Neurolucida software (MBF Bioscience, Williston, VT, USA) and refined with Adobe Illustrator (Adobe Systems, Inc., San Jose, CA, USA). Photomicrographs were captured using either a Zeiss AxioImager Z1 fluorescence microscope and AxioCam HRm or HRc cameras (Zeiss) controlled by AxioVision Software (version 4.6, Zeiss) or with structured illumination microscopy on the AxioImager Z2 as described above. Final images were produced by selecting the relevant optical sections from the stack and creating a maximum projection image (Neurolucida 11.03 software). Electron micrographs were assembled from the digital files. For both light and electron micrographs, Adobe Photoshop (Version CS3 or CS6, Adobe Systems) was used to add labels, crop images, erase background around tissue sections and to colorize monochrome images. Contrast levels were adjusted globally by adjusting levels or curves.

## Results

### Identification of the NBIC in guinea pigs

The NBIC in guinea pigs was identified by comparison with descriptions in cats, ferrets and rats (Kudo et al., 1984; Schnupp and King, 1997; Paxinos and Watson, 1998). The rostral end, where the NBIC abuts the MG, and the caudal end, where the NBIC abuts the IClc, can be difficult to delineate in standard Nissl stains. We report here that bNOS and GAD immunostaining are particularly helpful in these areas. At the caudal end, the NBIC has less intense bNOS staining and less intense GAD staining than the IClc, allowing the structures to be distinguished. The rostral borders were particularly enhanced by the anti-GAD staining. The MG in guinea pigs is nearly devoid of GABAergic cells (Arcelli et al., 1997). GABAergic cells are present throughout the NBIC, distinguishing it from the MG. GAD+ puncta (i.e., boutons) also differ, being much more numerous in the MG than in the NBIC.

### NBIC projections to the MG

**Figure 1:**
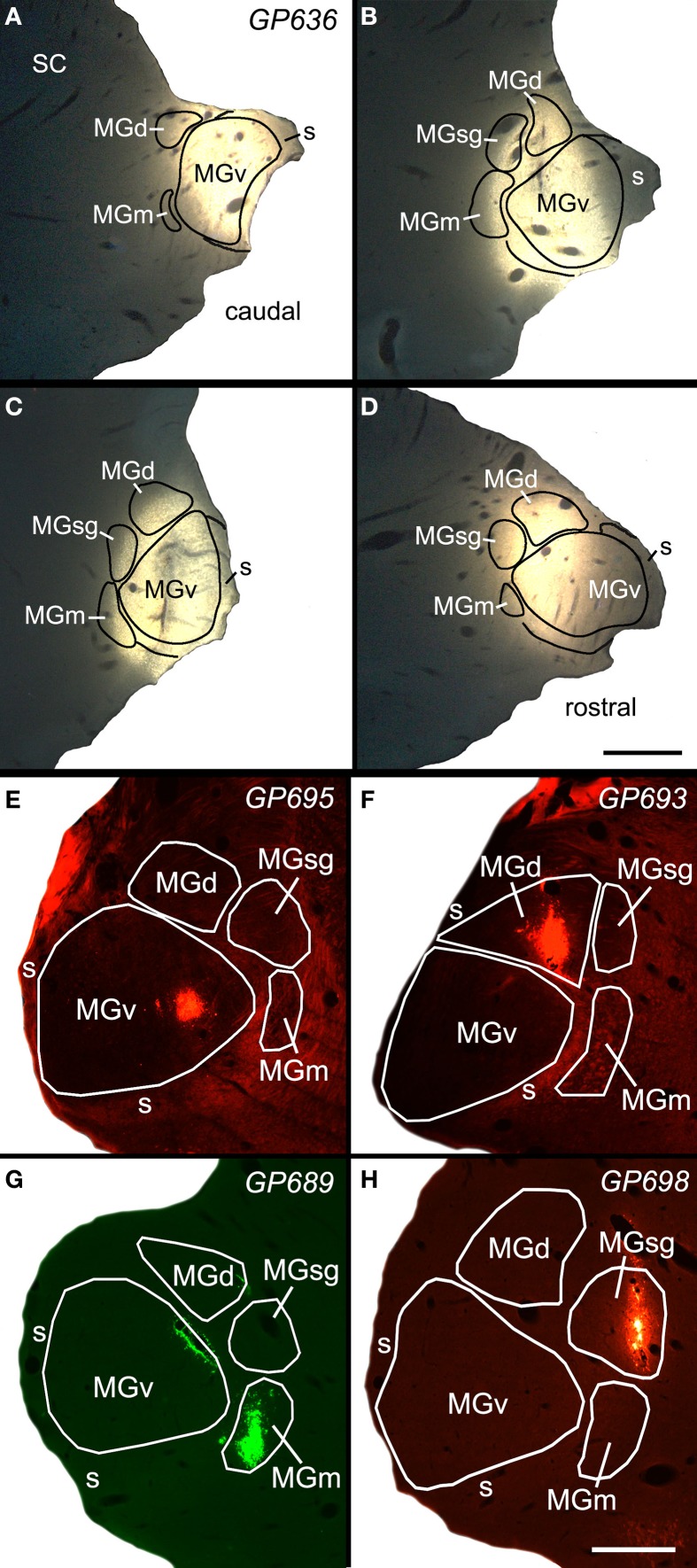
**Photomicrographs of a representative large injection site (A–D) and 4 small injection sites (E–H) in the medial geniculate nucleus (MG)**. **(A–D)** Series of transverse sections through the MG showing a large injection of FluoroGold that involved all MG subdivisions. Sections are arranged from caudal to rostral. Scale bar in D = 1 mm. **(E)** Small injection of red beads confined to the ventral subdivision of the MG (MGv). **(F)** Small injection of red beads confined to the dorsal subdivision of MG (MGd). **(G)** Small injection of green beads into the medial subdivision of the MG (MGm). Additional green fluorescence is seen around the margins of a blood vessel along the dorsomedial border of the ventral MG (v); this represents spread of beads that does not result in retrogradely labeled cells. This injection was into the right MG; the image has been reversed left to right to facilitate comparisons with the other small injections. **(H)** Small injection of red beads into the suprageniculate MG (sg). Scale bar in H = 0.5 mm and applies to **(E–H)**. Transverse sections; dorsal is up; lateral is to the left in **(A–D)**, and to the right in **(E–H)**. Panels **(A–D)** were previously published in Mellott et al. (2014); panel **(G)** was previously published in Schofield et al. (2014b).

Figure 1 shows a representative example of a large injection of FluoroGold (FG) into the right MG. This injection included parts of all 4 major subdivisions (ventral, dorsal, medial, and suprageniculate) as well as the MG shell. The injection did not spread to the caudal border of the MG, and completely missed the caudal end of the MGm, which abuts the NBIC. We conclude that the injection did not encroach directly on the NBIC. Retrogradely labeled cells were very numerous in many regions, including the ipsilateral and contralateral IC and many subcollicular auditory nuclei previously described as sources of input to the MG (Anderson et al., 2006; Schofield et al., 2014a,b) and in the NBIC. The following description is limited to label in the NBIC. As described above, the NBIC consists of a concentration of cells located medial to the main bundle of inferior brachium fibers and cells located among these fibers. Following tracer injections in the MG, labeled cells were quite numerous within the large nuclear region and scattered in smaller numbers within the fiber tract. The majority of labeled cells in the NBIC were located ipsilateral to the injection site, although a few labeled cells were present contralaterally.

**Figure 2:**
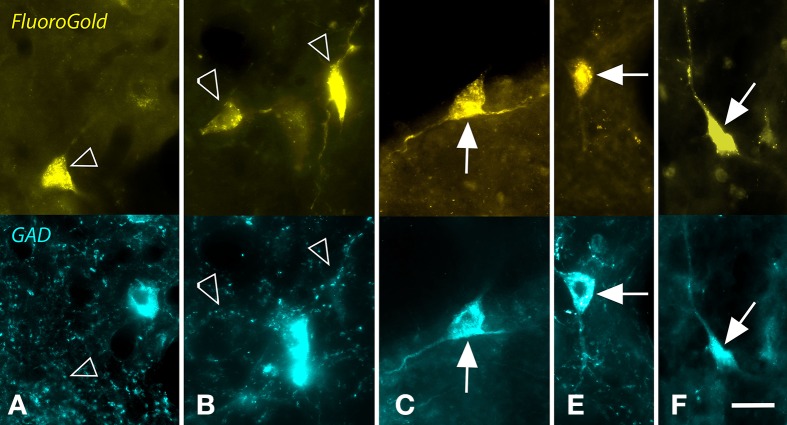
**Photomicrographs showing examples of GAD-negative and GAD+ cells in the nucleus of the brachium of the inferior colliculus that were retrogradely labeled by a large injection of FluoroGold (FG) into the ipsilateral medial geniculate nucleus**. For each panel, the upper image shows cells labeled with FG and the lower image shows the same region visualized for GAD immunoreactivity. **(A,B)** Examples of FG-labeled cells (open arrows) that were GAD-negative. In both panels, nearby cells were robustly labeled for GAD (blue cells). **(C–F)** Examples of FG-labeled cells (solid arrows) that were also GAD+. Scale bar = 20 μm. **A,C–E**: GP636; **B**: GP721.

**Figure 3:**
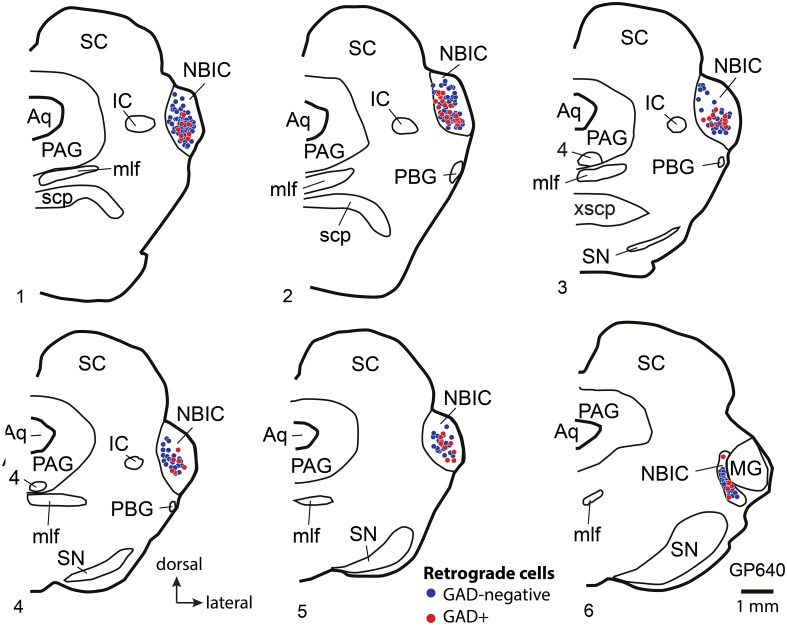
**Plots showing the distribution of labeled cells in transverse sections through the right nucleus of the brachium of the inferior colliculus (NBIC) after a large injection of Fast Blue (FB) into the right MG**. Each symbol represents one retrogradely-labeled cell. Symbol color indicates whether the FB-labeled cell was GAD-negative (blue circle) or GAD+ (red circle). Sections are numbered from rostral to caudal and are spaced 300 μm apart. See list for abbreviations.

A proportion of the retrogradely labeled cells were immunopositive for GAD (GAD+) (Figure 2), while the remaining cells were GAD-negative. The two cell types were intermingled, with no obvious relationship to the location of the injection site within the MG. Although negative staining can be difficult to interpret, retrogradely labeled immunonegative cells were often located near GAD+ cells (e.g., Figures 2A,B). Such a result suggests that the immunonegative cell was in fact non-GABAergic, and not unlabeled because of incomplete immunostaining. Figure 3 shows a plot of the labeled cells in the right NBIC following a large injection of Fast Blue into the right MG. Labeled cells were located throughout the rostro-caudal length of the NBIC, extending from the caudal end of the nucleus where it abuts the lateral cortex of the IC to the rostral end at the level of the MG. The majority of cells, however, were concentrated between these two extremes, i.e., in transverse sections with a prominent superior colliculus. GAD+ retrogradely-labeled cells (red symbols in Figure 3) were located throughout the rostro-caudal extent of the NBIC.

The proportion of retrogradely labeled NBIC cells that were GAD+ was quantified from 3 experiments with large injections into the MG (Table 3, top panel). On average, 17% of the retrogradely labeled cells were GAD+. The values ranged from 14 to 21% across the 3 experiments (490 cells counted). Some of the variability may reflect the 3 different retrograde tracers (FB, FG, and RB) but a second possibility is that the projections (GABAergic or otherwise) do not terminate evenly across the MG subdivisions. Analysis of results from 7 experiments with small injections support this latter interpretation. Two injections confined to the MGv (GP695, GP719) produced almost no labeled cells in the NBIC and were not analyzed further. Five additional injections labeled enough cells in the NBIC to warrant quantification (Table 3, bottom panel) Injections into the MGm resulted in 13–14% GAD+ retrograde cells, whereas injections into the MGsg resulted in only 2–4% GAD+ retrograde cells. Injections into the MGd labeled an intermediate amount (9% of retrograde cells were GAD+).

**Table 3:** **Percentage of retrogradely labeled NBIC cells that are GAD-immunopositive (GAD+)**.

**Case**	**Tracer**	**# Sections**	**MG subdivisions**	**Retro only**	**Retro-GAD+**	**% GAD+**
**LARGE INJECTIONS**						
GP636 R	FG	3	all	85	14	14%
GP640 L	RB	7	all	92	18	16%
GP640 R	FB	7	all	221	60	21%
Totals				398	92	
Averages				133	31	17%
**SMALL INJECTIONS**						
GP689 L	RB	4	MGm/sg	49	7	13%
GP689 R	GB	4	MGm	66	11	14%
GP696 L	RB	5	MGsg	52	1	2%
GP698 L	RB	6	MGsg	94	4	4%
GP718 L	FG	5	MGd	103	10	9%
Totals				364	33	
Averages				73	7	8%

Summary of GAD+ and GAD-negative (“Retro only”) labeled cells in the NBIC after large injections of retrograde tracer into the MG (upper panel) or small injections into 1 or 2 MG subdivisions. Note that 2 animals with injections confined to the MGv were included for study (see Table 1), but had so few labeled cells in the NBIC that they were not included in the quantitative analysis. “Case” indicates the experiment number and the side of the tracer injection. # sections, number of sections in which retrogradely labeled cells were counted. Note that cells were counted only at depths within the sections that were well-stained with the GAD immunomarker (see Materials and Methods). Retro only, number of cells that were labeled with the retrograde tracer and were GAD-immunonegative. “Retro-GAD+” = number of cells that were retrogradely labeled and were also GAD-positive. “% GAD+” = percentage of retrogradely labeled cells that were GAD, calculated as the number of Retro GAD/(Retro only + Retro-GAD). FB, Fast Blue; FG, FluoroGold; RB, red beads.

### Auditory cortical projections to NBIC

**Figure 4:**
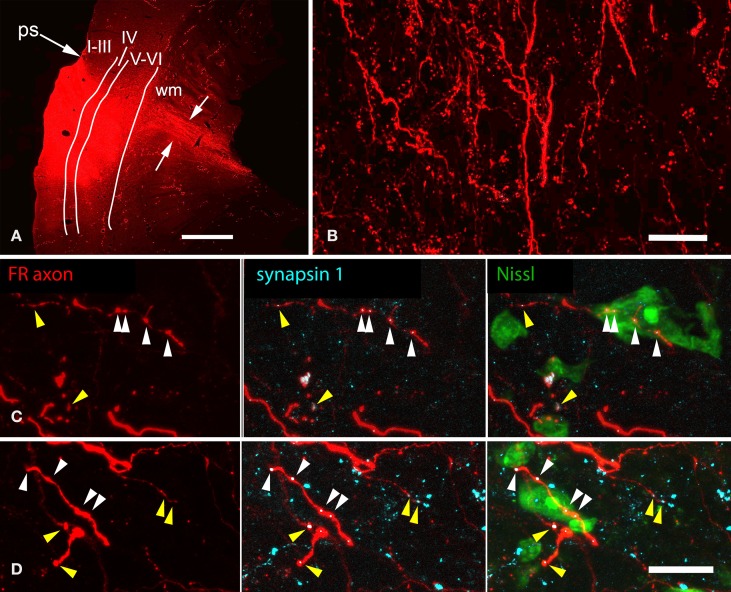
**Photomicrographs showing representative results after an injection of FluoroRuby (FR) into the left auditory cortex**. **(A)** Transverse section showing part of a large deposit of FR into the left auditory cortex. The deposit site (bright red area) was confined to the cortical layers (indicated by Roman numerals); FR-labeled axons (between white arrows) are seen extending into the subcortical white matter (wm). GP724. ps, pseudosylvian sulcus. Dorsal is up; lateral is to the left. Scale bar = 1 mm. **(B)** FR-labeled axons in the nucleus of the brachium of the inferior colliculus (NBIC) after the injection shown in panel **(A)** labeled axons as well as many boutons are visible. Sagittal section. Scale bar = 20 μm. Structured illumination image stack; maximum projection image. **(C,D)** Structured illumination fluorescence images showing FR-labeled boutons (red) in the NBIC that are also immunoreactive for the synaptic marker synapsin 1 (imaged with AlexaFluor 647 and colorized cyan). Each row shows a single area visualized for FR (all panels), synapsin 1 (cyan, middle and right panels) and Neurotrace (a Nissl stain, colorized green, right panel). Many swellings on the FR-labeled axons were immunopositive for synapsin 1; some of these were closely apposed to labeled somas (white arrowheads), whereas other synapsin 1-positive, FR labeled boutons were located in the neuropil (yellow arrowheads), between Nissl-stained cells (presumably forming synapses with unlabeled dendrites). GP724. Sagittal sections. Scale bar = 20 μm.

Multiple areas of the AC project to the NBIC (Saldaña et al., 1996; Winer et al., 1998; Kimura et al., 2004; Budinger et al., 2006), so we injected anterograde tracer at multiple sites across temporal cortex. Figure 4A shows a representative injection site of FR into the left AC. The deposits typically spanned multiple cortical layers, including layers V and VI, but did not extend below layer VI into the white matter. Tangentially, the deposit sites spanned a wide region of temporal cortex, including regions adjacent to the pseudosylvian sulcus, a surface landmark near the border between A1 and adjacent auditory belt areas S (“small area”) and dorsorostral belt (cf. Wallace et al., 2000, 2002). We conclude that the injection sites labeled corticofugal axons originating from multiple subdivisions of the AC, including primary AC (A1) and (across cases) varying amounts of the surrounding auditory areas. Labeled axons and boutons were present in large numbers in numerous subcortical nuclei, including the ipsilateral MG, ipsilateral and contralateral IC and other brainstem nuclei as described in previous studies (e.g., Feliciano et al., 1995; Coomes and Schofield, 2004; Coomes Peterson and Schofield, 2007; Schofield and Motts, 2009). The present report focuses on labeled axons in the NBIC.

On the side ipsilateral to the cortical injection, the brachium of the IC (i.e., the fiber pathway itself) contained many labeled axons (Figure 4B). No labeled axons were found in the contralateral NBIC, so the remaining discussions are limited to the ipsilateral NBIC. Many of the axons labeled in the ipsilateral NBIC presumably continue on to the IC, one of the most prominent targets of corticofugal projections. Nonetheless, labeled axons also give rise to boutons within the brachium, including terminal boutons as well as en passant boutons. Some of these axons arose from collateral branches of axons that continued caudally (perhaps to reach the IC). It was not possible to determine if some axons terminate in the NBIC without additional branching. While it is typical for boutons to represent presynaptic sites, the presence of synapses within the NBIC was of particular concern given the large number of passing axons. We first labeled synaptic sites with antibodies to synapsin 1, a molecule involved in linking synaptic vesicles to the cytoskeleton and thus concentrated in presynaptic terminals. Figures 4C,D show examples of FR-labeled boutons that also show punctate labeling with synapsin 1. The penetration of the anti-synapsin was rather limited, so only a portion of the tissue near the surface could be analyzed. Nonetheless, double-labeled boutons were readily observed. The majority of such boutons (88%; 399/455 boutons analyzed) were located in the neuropil, presumably to contact dendrites of NBIC cells (Figures 4C,D, yellow arrowheads). We also observed double-labeled boutons (12%) in apparent contact with NBIC cell bodies (Figures 4C,D, white arrowheads).

**Figure 5:**
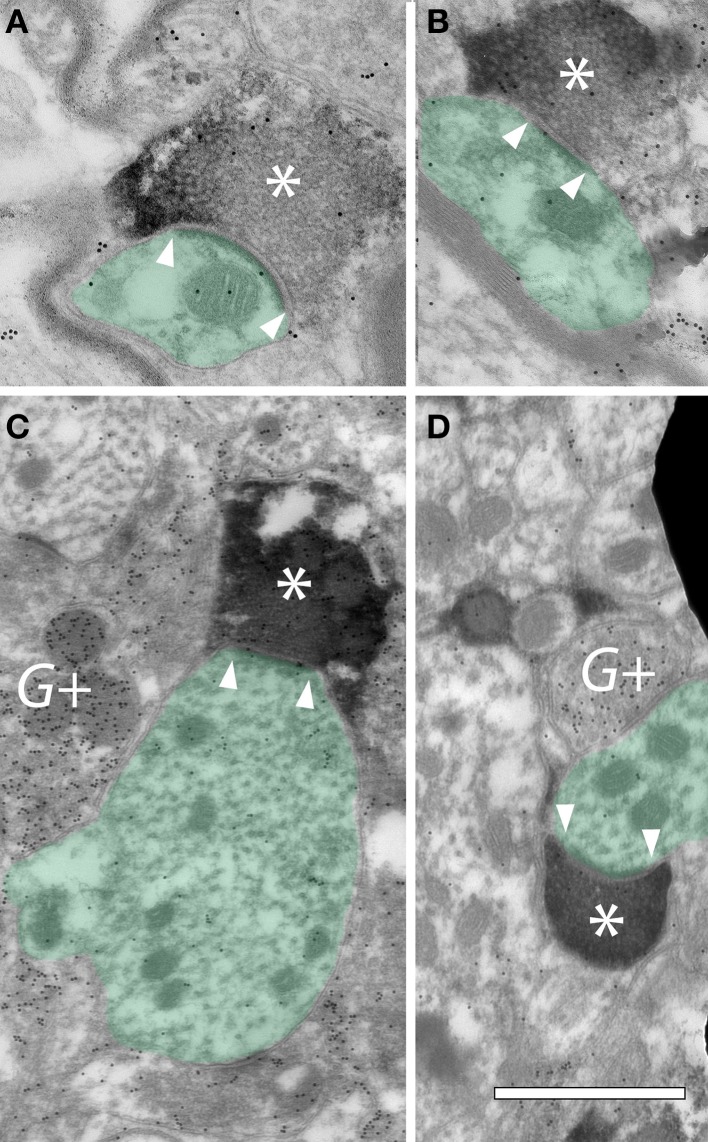
**Electron micrographs showing FR-labeled cortical boutons forming synapses in the ipsilateral nucleus of the brachium of the inferior colliculus (NBIC)**. **(A–D)** Cortical boutons (^*^) are filled with DAB precipitate resulting from transport of FluoroRuby. Internal structures in the boutons were obscured by the DAB precipitate, but round synaptic vesicles could often appear negatively stained within the profiles. Postsynaptic densities were typically prominent (located between the white arrowheads). The postsynaptic targets of these cortical boutons were dendrites (green shading). Post-embedding immunogold staining was used to identify GABAergic profiles, indicated by gold particles (small black dots) (G+). GP363. Scale bar = 1 μm.

To further establish the presence of cortical synapses, we used electron microscopy to examine NBIC tissue in animals in which AC axons were labeled with FR and subsequently made electron dense with diaminobenzidine (DAB). GABAergic neurons were also labeled with antibodies to GABA. The latter staining was visualized with immunogold particles that are readily distinguished from the DAB label that identified the cortical boutons. Figure 5 shows examples of DAB-labeled cortical boutons forming synapses on dendrites in the NBIC. The results confirm that cortical axons form synapses with NBIC cells. The cortical boutons were generally small, with profile diameters on the order of 0.5–1.0 μm (Figure 5). Prominent postsynaptic densities, indicating asymmetric synapses, ranged from 0.25 to 0.5 μm in length and could be straight or curved (concave toward the bouton) (Figure 5). The synaptic vesicles were round and very numerous, often filling much of the presynaptic profile. Our small sample of synapses contacted dendrites (Figure 5), which is consistent with our light microscopic data showing a majority of cortical boutons ending in the NBIC neuropil. While GABA+ profiles were sometimes in close proximity to the cortical boutons (Figures 5C,D), the postsynaptic profiles of the cortical boutons were GABA-negative. A larger sample size would be needed to characterize contacts on NBIC somas and GABAergic cells.

### Auditory cortical projections to NBIC cells that project to the MG

**Figure 6:**
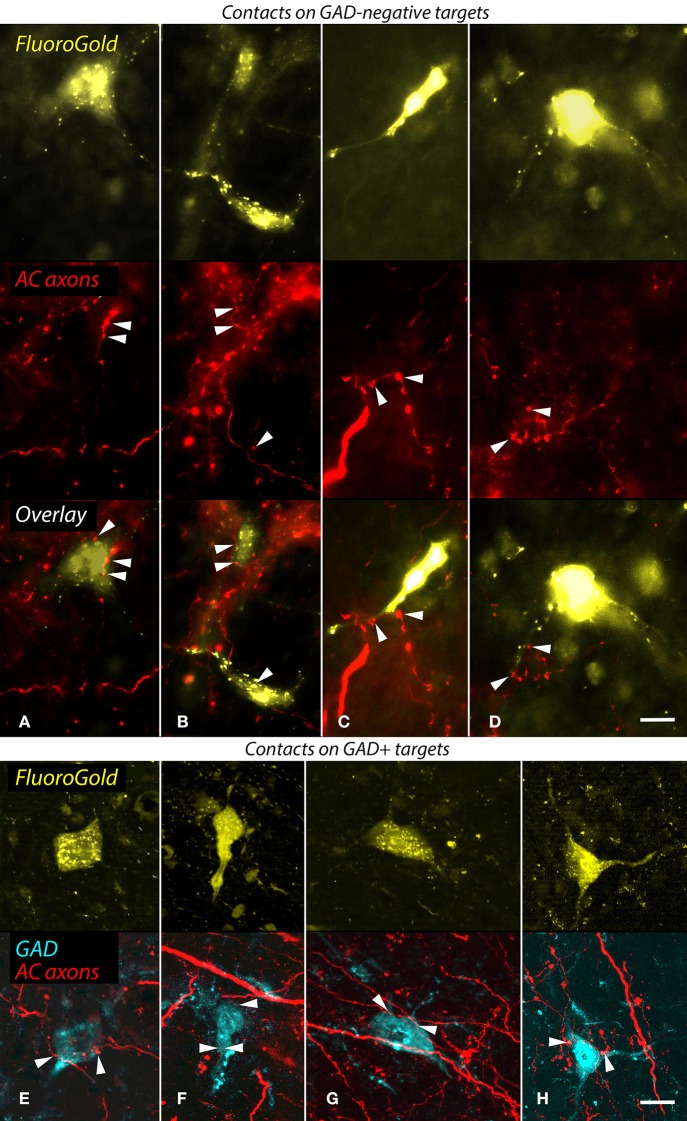
**Structured illumination fluorescence micrographs showing FluoroGold (FG)-labeled GAD-negative and GAD+ cells in the nucleus of the brachium of the inferior colliculus (NBIC) that project to the medial geniculate nucleus (MG) and appear to receive synaptic inputs from FluoroRuby (FR)-labeled auditory cortical (AC) axons**. **(A–D)** Cortical contacts on GAD-negative cells. In each column, the top image shows NBIC cells labeled by an injection of FG into the ipsilateral MG. The middle panel shows the same area visualized for axons labeled by an injection of FR into the ipsilateral AC. The bottom panel shows an overlay of the images, with labeled AC boutons (arrowheads) in close apposition to FG-labeled cell bodies **(A,B)** or proximal dendrites **(C,D)**. The cells in **(B,D)** were labeled by an injection restricted to the dorsal MG (MGd; GP718); the cell in **(A)** was labeled by a large injection into all MG subdivisions (GP721), and the cell in **(C)** was labeled by an injection into the dorsal and ventral MG (MGd, MGv; GP726). Scale bar = 10 μm. **(E–H)** Apparent AC contacts onto GAD+ NBIC cells. The upper images in each column show FG-labeled NBIC cells after FG injections into all MG subdivisions (**E,G,H**; GP721) or an injection into the medial (MGm) and suprageniculate (MGsg) (**F**; GP722). The lower panel in each column shows the overlaid images from the same area depicting GAD immunostaining (colorized cyan) and FR-labeled cortical axons (red). All four FG-labeled cells are also GAD-immunoreactive (GAD+). In addition, FR-labeled boutons are in apparent contact (arrowheads) with the somas or proximal dendrites of each labeled cell. Structured illumination fluorescence; “maximum projection” images from image stacks (optical section thickness = 0.2 μm). Scale bar = 10 μm.

As described in the Introduction, the NBIC projects to numerous targets. Our final question concerned the projections of the NBIC cells contacted by cortical axons. Specifically, we asked whether any of the cortically-targeted NBIC cells project to the MG We addressed this question with structured illumination fluorescence microscopy and a combination of anterograde labeling of auditory cortical axons, retrograde labeling of NBIC cells that project to the MG, and immunostaining with anti-GAD to label GABAergic cells. Every case with successful labeling of both anterograde and retrograde pathways provided evidence for cortical contacts onto NBIC-MG cells. Figures 6A–D show examples of cortical boutons in contact with GAD-negative NBIC cells labeled by retrograde transport of FluoroGold from the MG. The cortical axons appeared to contact both somas and proximal dendrites. Other FR-labeled cortical boutons appeared to contact GAD-positive NBIC cells that project to the MG (Figures 6E–H). Once again, the boutons appeared to contact both somas and proximal dendrites. In addition, there were many labeled cortical boutons located in the neuropil without any obvious association with retrogradely labeled cells. The retrograde tracers rarely labeled the dendrites extensively, so it is possible that cortical boutons also contact the distal dendrites of NBIC cells that project to the MG. However, it is also possible that these boutons contact NBIC cells that were not labeled by the tracer either because the injection did not fill the entire MG or because the contacted cells project to some other target (e.g., the superior colliculus, a prominent target of NBIC projections).

## Discussion

The present results demonstrate a projection from the NBIC to the MG in guinea pigs that originates from both GAD+ and GAD-negative cells. The GAD+ cells constitute ~17% of the projecting population. Below, we discuss evidence that the GAD-negative NBIC cells are likely to be glutamatergic. Our data also demonstrate a projection from the AC to the NBIC similar to that described in numerous species (cat: Diamond et al., 1969; rat: Saldaña et al., 1996; Kimura et al., 2004; gerbil: Budinger et al., 2006). We identify cortical synapses in the NBIC and provide evidence that cortical axons contact both GABAergic and non-GABAergic (presumably glutamatergic) NBIC cells that project to the MG. These results suggest that corticofugal projections can modulate activity in both excitatory and inhibitory ascending projections from the NBIC to the auditory thalamus.

### Technical considerations

#### Tracer injections

In addition to dense projections to the MG, the NBIC projects to some surrounding nuclei (e.g., subparafascicular nucleus) as well-more rostral thalamic nuclei (e.g., paraventricular nucleus, Kudo et al., 1984). The course of NBIC axons to these extra-MG targets is not clear, so we cannot rule out the possibility that some of the retrogradely labeled cells in the present paper send axons to terminate in these other areas. Such labeling would be most likely in cases with large injections, where the microsyringe needle causes damage that could lead to labeling fibers of passage. However, all our findings were confirmed in cases with small injections made with micropipettes. This approach is much less likely to label fibers of passage, so we conclude that the majority of cells labeled in the present cases project to the MG.

Injections of tracer into the AC were designed to maximize labeling of corticofugal axons and thus the opportunities for finding connections with the retrogradely labeled NBIC cells. The injections appeared to be completely confined to AC (as described by Wallace et al., 2000, 2002), but we are unable to distinguish contributions from different cortical areas. Studies in cats and rats suggest that multiple AC areas project to the NBIC (Diamond et al., 1969; Andersen et al., 1980; Saldaña et al., 1996; Kimura et al., 2004), and one might predict that projections from different areas serve different functions. Future studies will be needed to examine this issue.

#### Immunostaining and quantification

The anti-GAD antibody used for light microscopy in the present study has been verified previously in guinea pigs and is likely to have labeled the majority of GABAergic cells and few if any other cells (Nakamoto et al., 2013c). The staining was robust in that immunopositive cells were readily distinguished from immunonegative cells. As described in Materials and Methods (Section Light microscopy—brightfield and wide-field fluorescence), our quantitative analyses were limited to tissue depths that had robust immunostaining. We conclude that false negative staining, in which retrogradely-labeled GABAergic cells were unstained by the immunomarker, is unlikely to have affected our results substantially. The anti-GABA antibody used for EM has also been validated in guinea pigs (Nakamoto et al., 2013a) and is likely to have labeled the majority of GABAergic profiles.

The percentage of retrogradely labeled cells that were GAD+ varied across cases. To some extent this can be related to MG subdivision, but there may also be differences due to the tracers. The percentages after small injections (2–14%) never reached as high values as those obtained after large injections (14–21%). It is possible that GABAergic NBIC projections are particularly dense to small parts of the MG that were included in our large injection sites but not our small injections. A second possibility is that the GABAergic cells are less efficiently labeled by the tracers (perhaps they have fewer axonal terminals than the glutamatergic cells) and thus are less well-labeled by smaller injections. While we cannot rule out such issues, the values of 13–14% for the GABAergic component of projections to the MGm is quite close to the values reached in 2 of the 3 large injections (14, 16, and 21%; see Table 3).

The other antibodies used here have been characterized previously. We used concentrations of antibodies that we have optimized empirically for guinea pigs and for the current fixation protocol, so we expect false negatives and/or false positives to be minimal. The use of bNOS was limited to cytoarchitectural analysis; our results matched those published in guinea pigs (Coote and Rees, 2008) and are unlikely to have affected our conclusions. Anti-fluorescein and anti-rhodamine staining was absent when applied to tissue that did not have tracer injections, so we are unlikely to have any substantial artifactual staining with these antibodies. The synapsin antibody was validated by Western blot (by the manufacturer). As described in the Results, the penetration of this antibody was limited so false negatives would be prominent in deeper parts of the sections. We avoided this area by restricting our analysis of synapsin staining to tissue near the cut surfaces (where staining was prominent).

#### Limitations of light and electron microscopy

Light microscopy with fluorescent markers allows us to survey a large area of tissue while visualizing multiple markers, but is limited in its ability to identify synaptic contacts. The latter issue is addressed by electron microscopy (EM), but technical requirements of EM limit the number of distinguishable labels and the amount of tissue that can be analyzed in a reasonable time. By combining the two approaches, we obtained several types of information regarding the projection of the AC to the NBIC. Our results with light microscopy provide strong evidence for cortical synapses in the NBIC. The electron microscopy results support this conclusion. Our small sample of synapses analyzed with EM did not reveal GABAergic targets of AC boutons. While we did not quantify the GAD+ vs. GAD-negative targets in our light microscopy data, the proportion of apparent targets that were GAD+ were clearly a minority. In fact, our retrograde tracing showed that a minority (21% or less) of the NBIC-MG cells are GAD+, so cortical boutons would have to be highly biased toward GABAergic cells for them to represent a large proportion of the targets. We conclude that AC boutons are likely to contact NBIC GABAergic cells but that the frequency of such contacts is too low to have been revealed in our small EM sample. Fluorescence microscopy also facilitates multi-labeling experiments. Our triple-label experiments, combining anterograde and retrograde tracing with immunochemistry, suggest that some of the NBIC cells contacted by AC axons project to the MG, and that this includes both GABAergic and non-GABAergic cells. Future experiments with triple labeling adapted for EM will be needed to confirm cortical synapses with NBIC GABAergic cells as well as cortical synapses on MG-projecting NBIC cells.

### Functional implications

#### Physiology of NBIC-MG projections

*In vitro* experiments demonstrate that electrical stimulation of the brachium of the IC leads to both excitation and inhibition of MG cells (e.g., Hu et al., 1994; Peruzzi et al., 1997; Bartlett and Smith, 1999; Smith et al., 2007). The results of such experiments are generally interpreted in terms of activating IC inputs to the MG, but it seems likely that NBIC projections to the MG are also stimulated, either by direct stimulation of the NBIC cells or by stimulation of the NBIC axons that intermingle with IC axons in the brachium as the brachium nears the MG (Kudo and Niimi, 1980; Kudo et al., 1984). The effects of brachial stimulation on MG cells are blocked by antagonists of GABA and glutamate, indicating that the ascending fibers in the brachium use these two transmitters. It is likely, then, that the non-GABAergic cells in the present study are glutamatergic and that the NBIC, like the IC, provides both glutamatergic and GABAergic ascending inputs to the MG.

A previous study identified GAD-positive cells in NBIC that project to the MG in rats (Peruzzi et al., 1997). The present study identified a higher percentage of GABAergic cells (17 vs. 10%), but in both species the GABAergic cells form a minority of the projection. How this inhibition is integrated with other projections to the MG remains to be determined. Using *in vivo* intracellular recording in guinea pigs, Yu et al. (2004) showed that acoustic stimulation could elicit excitation and inhibition in MG cells. Some cells showed a combination of excitation and inhibition, while others showed only excitation or only inhibition. While the source of the inhibition could not be specified (the MG receives ascending GABAergic inputs from the IC as well as from the NBIC), the data indicate that inhibitory inputs can be driven by acoustic stimuli and may dominate the responses of some MG cells in some instances. Smith and colleagues have used *in vitro* recording to investigate the ascending excitation and inhibition to the MG (Bartlett and Smith, 1999; Smith et al., 2006, 2007). By electrically stimulating the brachium of the IC, they distinguished populations of MG cells that receive only excitatory inputs, only inhibitory inputs or convergent excitatory and inhibitory inputs. The populations show different integrative properties suggesting different functions in hearing. The authors suggested that early inhibition could prevent responses to later excitatory inputs (thus gating the responses of MG cells) whereas later inhibition could alter the onset/sustained nature of responses or modify response selectivity for temporal properties of a stimulus. Thus, both *in vivo* and *in vitro* recordings suggest that ascending inhibitory inputs could play important, and multiple, roles in the responses of MG cells.

#### NBIC projections in general

The functions of NBIC projections to the MG remain elusive. NBIC has been studied more for its connections with the SC and a role in orientating responses (e.g., Redgrave et al., 1987; King et al., 1998; Doubell et al., 2000). NBIC cells can show tuning for spatial cues and direction of movement (Aitkin and Jones, 1992; Schnupp and King, 1997; Slee and Young, 2013). It is not known if the same NBIC cells project to the SC and the MG, but it appears reasonable to expect that NBIC projections to the MG may play a role in spatial hearing, orientation and, perhaps, auditory attention.

Further insights may be gained by considering the subdivisions of MG that are targeted by NBIC axons. NBIC projects to the extralemniscal MG, largely avoiding the MGv but terminating widely to include MGd, MGm, and MGsg as well as surrounding areas (and several intralaminar nuclei of the thalamus) (present data; Kudo et al., 1984). These areas serve numerous functions. Anderson and Linden (2011) suggested a role in detecting stimulus change and analyzing stimulus context. Numerous studies have associated these areas with behavioral cueing, whereby auditory or other sensory inputs can be associated with positive or negative reinforcement (reviewed by Hu, 2003). Projections from these areas to the amygdala appear critical for such associations (LeDoux et al., 1985). Finally, these areas of the MG also project to the basal ganglia and may support orienting responses or could be involved in sensory gating and selection among multiple stimuli. One or more of these functions might be considered for the NBIC.

### AC projections to NBIC

Projections to the NBIC originate from primary AC, the suprasylvian fringe (in cats) and the posterodorsal area (in rats) (Diamond et al., 1969; Andersen et al., 1980; Saldaña et al., 1996; Winer et al., 1998; Kimura et al., 2004; Budinger et al., 2006). The laminar origins of these projections are unknown. Saldaña et al., (1996) described boutons in the NBIC arising from branches of corticocollicular axons. Corticocollicular projections arise from layer V and, to a lesser extent, from layer VI (Schofield, 2009), suggesting that these layers are the source(s) of projections to NBIC.

Round synaptic vesicles and asymmetric synaptic junctions (present data) suggest that the cortical projections to NBIC are excitatory. The present data suggest that both GABAergic and non-GABAergic NBIC cells are contacted by AC axons. If these cells can be driven by the AC inputs, then the corticofugal system could elicit both excitation and inhibition in the targets of the NBIC cells. Such a dual effect has been attributed to AC projections to the IC, where physiological studies have identified direct excitation of collicular cells as well as indirect inhibition (and excitation) of collicular cells following stimulation of the AC (Mitani et al., 1983). These effects have been attributed to AC axons forming excitatory synapses with glutamatergic and GABAergic collicular cells that, once activated by the cortical inputs, subsequently excite or inhibit other collicular cells via local axon collaterals (discussed in Nakamoto et al., 2013b). NBIC cells appear to have local axons in addition to extrinsic projections (Ramón y Cajal, 1995), so cortical inputs may affect NBIC cells directly and via local connections.

### Corticofugal projections and subcortical auditory circuits

The corticofugal system has been implicated in the selectivity of subcortical cells for specific stimulus parameters and the plastic retuning of responses according to stimulus salience or altered sensory input (e.g., Jen et al., 1998; Popelár et al., 2003; Nakamoto et al., 2008, 2010; Suga, 2008; Xiong et al., 2009; Bajo et al., 2010; He and Yu, 2010; Anderson and Malmierca, 2013). Many studies have focused on projections to the IC or the MG, but corticofugal effects have been observed from the thalamus to the cochlea. Together, the cortical effects allow the auditory system as a whole to respond more effectively to stimuli of particular significance and to adapt to changes in sensory input. The circuitry that underlies these functions includes direct AC projections to the subcortical auditory structures as well as cortical connections with modulatory systems that affect all levels of the auditory pathway (e.g., Metherate, 2011; Schofield et al., 2011).

Many nuclei targeted by AC projections are themselves a source of projections to numerous places, so a key step for understanding the functions of AC projections to a given target is to identify the projections of targeted cells. Projections to the IC, for example, appear to contact multiple pathways, including ascending pathways to the MG (Coomes Peterson and Schofield, 2007), descending pathways to the CN (Schofield and Coomes, 2006), commissural pathways between the two colliculi (Nakamoto et al., 2013c) and intrinsic connections within the IC (Jen et al., 2001). Such pathways establish feedback loops that allow the AC to modulate its own input as well as multi-neuronal descending chains that extend the functional reach of the AC projections. Cortical projections to other targets may have more limited range. AC projections to the superior olivary complex contact some cells that project to the IC (Coomes Peterson and Schofield, 2007) and other cells that project to the cochlea (Mulders and Robertson, 2000), thereby affecting both ascending and descending pathways from the superior olive. However, not all output pathways from the superior olive are targeted by the AC; the medial superior olivary nucleus, a major source of binaural inputs to the IC, receives virtually no AC inputs. The cochlear nucleus is another area where AC projections contact only a subset of the potential targets. AC axons contact granule cells (whose projections are contained entirely within the cochlear nucleus) as well as cells that project to the IC (Weedman et al., 1996; Jacomme et al., 2003; Schofield and Coomes, 2005b). There is no evidence for cortical inputs to octopus cells, spherical bushy cells or globular bushy cells of the cochlear nucleus, suggesting that the output pathways from these cells are relatively insulated from cortical inputs. While many questions remain, the available evidence demonstrates that AC projections are in a position to have direct influence on selected brainstem circuits and thus to affect some auditory functions more than others.

The NBIC, despite being identified many years ago as a target of AC projections, has yet to be incorporated into a broad overview of the corticofugal system. The issue is relevant because of the wide array of projections from the NBIC, including ascending projections to the MG, to other thalamic nuclei and to the superior colliculus as well as descending projections to the IC (Kudo et al., 1983, 1984; King et al., 1998; Senatorov and Hu, 2002). Which of these output pathways from the NBIC are under direct influence by AC inputs? The present study provides a first step in identifying the ascending projections from the NBIC to the MG as a likely target of AC projections. This ascending projection comprises a large, presumably excitatory component and a smaller GABAergic component; both of these components appear to be contacted by cortical axons. Thus, the corticofugal projections are in a position to modify ascending excitatory and inhibitory projections from the NBIC to the thalamus. As suggested above, NBIC functions could involve sensory gating, selective attention or orienting responses and likely include integration across sensory systems. Identifying the roles of AC projections in such functions remain to be determined, and will require more information about the specific areas of the AC that project to the NBIC, the cell types that give rise to these projections, and the conditions under which these projections are active.

## Author contributions

Brett R. Schofield and Jeffrey G. Mellott designed and performed the experiments and analyzed data. Jeffrey G. Mellott and Martha E. Bickford performed the electron microscopy and analyzed the data. Brett R. Schofield wrote the paper with input from the other authors.

### Conflict of interest statement

The authors declare that the research was conducted in the absence of any commercial or financial relationships that could be construed as a potential conflict of interest.

## Abbreviations

4: trochlear nucleus
AQ: cerebral aqueduct
FB: Fast Blue
FG: FluoroGold
GAD: glutamic acid decarboxylase
GB: green beads
IC: inferior colliculus
MG: medial geniculate body
MGd: dorsal division of medial geniculate
MGm: medial division of medial geniculate
MGsg: suprageniculate division of medial geniculate
MGv: ventral division of medial geniculate
mlf: medial longitudinal fasciculus
NBIC: nucleus of the brachium of the inferior colliculus
PAG: periaqueductal gray
PBG: parabigeminal nucleus
ps: pseudosylvian sulcus
RB: red beads
s: shell of MG
SC: superior colliculus
SN: substantia nigra.
